# Pregnant Patient With Infrarenal Abdominal Aortic Aneurysm Successfully Treated With EVAR

**DOI:** 10.1016/j.jaccas.2025.105287

**Published:** 2025-10-01

**Authors:** Tomomi Ueda, Hiroki Yagi, Nana Akiyama, Naoya Akiba, Katsuyuki Hoshina, Hiroshi Akazawa, Norifumi Takeda, Norihiko Takeda

**Affiliations:** a Department of Cardiovascular Medicine, The University of Tokyo Hospital, Bunkyō, Japan; b Marfan Syndrome Center, The University of Tokyo Hospital, Bunkyō, Japan; c Department of Genomic Medicine, The University of Tokyo Hospital, Bunkyō, Japan; d Department of Obstetrics and Gynecology, The University of Tokyo Hospital, Bunkyō, Japan; e Department of Vascular Surgery, The University of Tokyo Hospital, Bunkyō, Japan; f The Institute of Medical Science, The University of Tokyo, Minato, Japan

**Keywords:** aortic aneurysm and dissection, endovascular aneurysm repair, genetic testing, pregnancy

## Abstract

**Background:**

Pregnancy management in patients with a history of untreated aortic aneurysm or dissection is not established.

**Clinical Condition:**

A patient with a chronic infrarenal abdominal aortic aneurysm and dissection was referred to our hospital at 13 weeks' gestation.

**Key Questions:**

What are the treatment options for infrarenal abdominal aortic aneurysm with dissection in a pregnant patient? What tests are needed for treatment strategy? What are the safety considerations when performing endovascular aneurysm repair (EVAR) during pregnancy?

**Outcome:**

Genetic testing for established hereditary aortopathy genes was negative. After carvedilol treatment, EVAR was performed under ultrasound guidance with minimal radiation exposure at 19 weeks' gestation, allowing for adequate fetal growth. She had an uncomplicated vaginal delivery under epidural anesthesia at 39 weeks' gestation.

**Take-Home Messages:**

EVAR could be an effective treatment option during pregnancy if radiation exposure is properly minimized. Multidisciplinary collaboration, including early evaluation for connective tissue diseases, is essential to evaluate treatment strategy.

## Graphical Abstract

**Figure:**
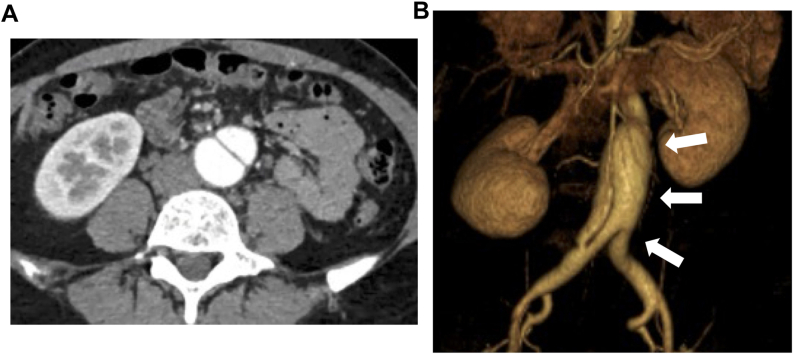

## Case Presentation

**Figure 1:**
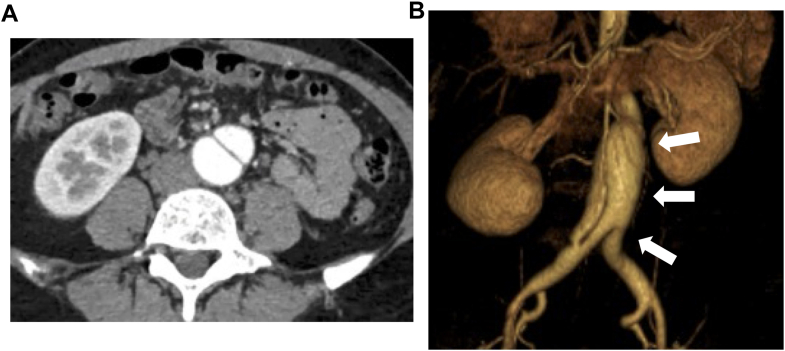
Incidental Infrarenal AAA and Dissection (A and B) Computed tomography scan showed 37 mm of infrarenal abdominal aortic aneurysm (AAA) complicated by dissection extending to the iliac artery; there was no evidence of atherosclerosis.

A 37-year-old Japanese woman with no past medical history was referred to our hospital at 13 weeks' gestation for the management of her pregnancy complicated by an incidental infrarenal abdominal aortic aneurysm (AAA) with dissection extending to the iliac artery. She was first diagnosed with asymptomatic infrarenal AAA 2 years earlier, when an abdominal ultrasound was performed during a routine workplace health check. A computed tomography (CT) scan confirmed infrarenal AAA with a maximum diameter of 36 mm complicated by dissection extending to the iliac artery. There was no evidence of atherosclerosis. Because she remained hemodynamically stable and free of end-organ malperfusion, the lesion was managed conservatively with imaging follow-up. CT scan obtained 1 year later demonstrated stability, with the maximum diameter measuring 37 mm (Figures 1A and 1B, Videos 1A to 1C). She had no past medical history including trauma or accident that might have complicated the aneurysm. She was a lifelong nonsmoker and consumed alcohol socially. Her family history was negative for aortic disease, connective tissue disease (CTD), or other heritable diseases. She worked as an office employee, and this was her first pregnancy.

**Take-Home Messages**

• EVAR could be an effective treatment option during pregnancy if radiation exposure is properly minimized.
• Multidisciplinary collaboration, including early evaluation for connective tissue diseases, is essential to evaluate treatment strategy.

On physical examination at presentation, she was 158 cm tall, weighed 49 kg, and had a body mass index of 19.6 kg/m^2^. Blood pressure was 110/62 mm Hg with a heart rate of 80 beats/min. A physical examination revealed no ectopia lentis, no musculoskeletal abnormalities including scoliosis and pectus carinatum, and no heart murmurs. However, an abdominal aortic pulse was palpable. No other physical findings suggestive of a CTD were observed. Laboratory results, including glycometabolic and lipid profiles, showed no abnormalities. Echocardiographic findings showed normal left ventricular ejection fraction, no dilatation of sinuses of Valsalva and ascending aorta, and no valvular disease.

## Clinical Question 1: What Are the Treatment Options for Infrarenal AAA With Dissection in This Patient, and Can the Pregnancy Continue With This Medical Condition?

The coexistence of CTD and hemodynamic, hormonal changes during pregnancy increases the risk of aortic dissection (AD).^1^ Therefore, therapeutic intervention for infrarenal AAA with dissection during pregnancy was deemed necessary, and several treatment options were considered, including open surgery and endovascular aneurysm repair (EVAR). Because of her relatively young age and lack of cardiovascular risk factors, hereditary CTD was considered a possibility. However, the risk of long-term complications from EVAR in patients with a CTD, including Marfan syndrome, Loeys-Dietz syndrome, and nonsyndromic hereditary thoracic aortic aneurysm and dissection, remains controversial.^2^ In addition, the outcomes of thoracic EVAR during pregnancy have been described in only a few cases, and it is not recommended in the presence of genetic aortopathy. Furthermore, women with vascular Ehlers-Danlos syndrome have a significantly higher risk of uterine rupture during pregnancy and delivery, and open surgery may not be a suitable option owing to vascular fragility and associated bleeding complications. Management of a pregnancy in the presence of these conditions can be extremely challenging.

## Clinical Question 2: Which Tests Should Be Added to Determine This Patient's Treatment Plan?

Recently, the discovery of several genes associated with thoracic aortic aneurysm and dissection has been accelerated by next-generation sequencing technology, and this trend has made genetic testing indispensable for the diagnosis of genetic diseases and their subsequent therapeutic management.^3^ Given the need for immediate assessment of vascular fragility to guide treatment of infrarenal AAA with dissection or the decision to continue pregnancy, the department of genomic medicine was consulted, and targeted gene panel testing for established hereditary aortopathy-related genes was conducted.

Genetic testing was approved by the Institutional Ethics Committee of the University of Tokyo, and the patient provided written informed consent. The panel included the following genes: *FBN1* (Online Mendelian Inheritance in Man [OMIM] ∗134797), *TGFBR1* (OMIM ∗190181), *TGFBR2* (OMIM ∗190182), *TGFB2* (OMIM ∗190220), *TGFB3* (OMIM ∗190230), *SMAD3* (OMIM ∗603109), *ACTA2* (OMIM ∗102620), *MYH11* (OMIM ∗160745), *MYLK* (OMIM ∗600922), and *COL3A1* (OMIM ∗120180). However, no pathogenic variants were identified, and the cause of infrarenal AAA with dissection remained unclear. At 18 weeks, she was informed of the result of the genetic test. Other CTDs, which were generally considered less likely to cause significant vascular damage than those described above, may also be present. Therefore, careful management and treatment were recommended.

## Clinical Question 3: What Strategy Should Be Used to Manage Infrarenal AAA With Dissection in This Pregnant Patient?

Tight blood pressure control, usually achieved with β-blockers, is recommended for aortic dilatation, previous dissection, or genetic predisposition. However, the routine prescription of β-blockers in these patients remains controversial. Renin-angiotensin system inhibitors are contraindicated in pregnancy because of teratogenicity. The European Society of Cardiology guidelines give a Class IIa, Level of Evidence: C recommendation for β-blocker therapy in pregnant patients with Marfan syndrome because its efficacy in preventing aortic root dilatation and dissection has not been conclusively demonstrated.^4^^,^^5^ In addition, a recent study showed a clear association between β-blockers and low birth weight, necessitating close fetal monitoring when these agents are used. In this case, carvedilol was started and titrated to 5 mg daily. After β-blocker initiation, maternal blood pressure and heart rate were adequately controlled, and fetal heart rate remained stable.

For acute Stanford type A AD in pregnancy, cesarean delivery of a viable fetus should precede aortic repair; if the fetus is not viable, repair is performed with the fetus in situ. For uncomplicated acute Stanford type B AD, close monitoring of the pregnant patient and fetus with conservative medical management is recommended.^4^^,^^5^ However, the optimal strategy for treating pregnant women with infrarenal AAA complicated by chronic dissection remains undetermined.

In this case, CT scan showed marked relative dilatation: the dissected/aneurysmal segment measured 37 mm, whereas adjacent native segments measured 16 mm in the mid-abdominal aorta and 15 mm in the “normal” infrarenal abdominal aorta—an approximate 2.3- to 2.5-fold enlargement. In addition, because only 2 pre-intervention imaging time points were available (initial diagnosis and a 1-year follow-up), aneurysm growth rate could not be reliably estimated, leaving behavior of the lesion under the physiologic stresses of ongoing pregnancy uncertain. This concern was heightened by persistent suspicion of an underlying CTD, which might predispose to rapid enlargement, new dissection, or rupture.

In the present case, through multidisciplinary discussions, we concluded that open surgery would be challenging considering adverse effects of postoperative intraabdominal adhesion on delivery and on the fetus owing to uterine compression during surgical manipulation. Meanwhile, the patient was found to be anatomically suitable for EVAR based on the following predefined criteria: proximal landing zone ≥15 mm below the renal arteries, aortic neck angulation ≤60°, iliac artery dilation ≤15 mm, and a distal iliac landing zone ≥10 mm, and thus EVAR was selected as the treatment strategy.

We elected to proceed after the completion of fetal organogenesis (ie, beyond the first trimester, ≥13 weeks) yet before advanced gestation to balance maternal aortic risk against fetal considerations. After genetic counseling, informed consent, and procedural planning, EVAR was performed at 19 weeks of gestation.

## Clinical Question 4: What Is the Maximum Safe Level of Radiation for Pregnant Women Undergoing EVAR?

**Figure 2:**
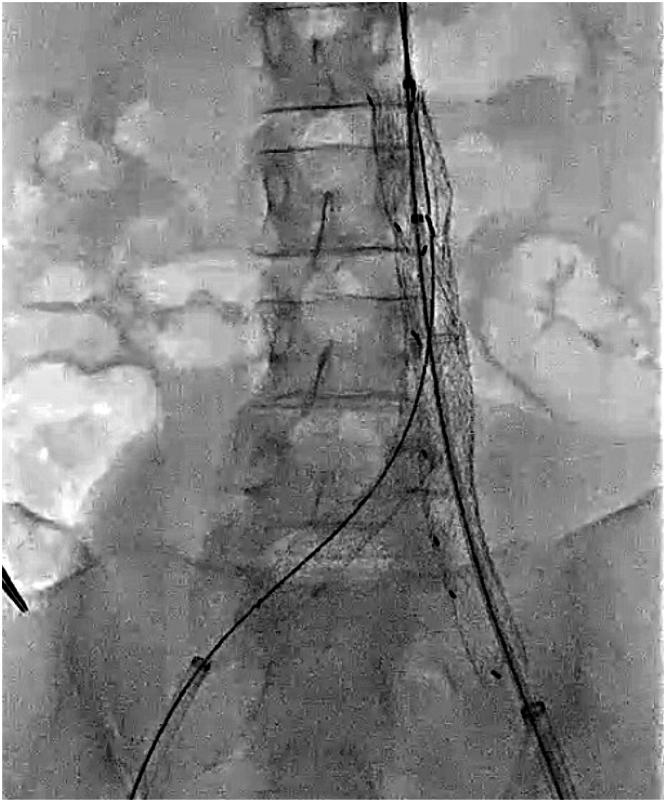
Endovascular Aneurysm Repair at 19 Weeks' Gestation A stent graft was successfully deployed from the abdominal aorta to both common iliac arteries without any complications at 19 weeks' gestation.

The mean dose area product in EVAR for AAA was 79.48 Gy cm^2^.^6^ Moreover, radiation exposure (cumulative air kerma) during pregnancy is generally considered safe if kept below 100 mGy; however, exposure above this threshold is associated with an increased risk of fetal harm.^7^ At 19 weeks' gestation, we performed EVAR (Figure 2). To minimize radiation exposure, we used transabdominal ultrasound to confirm the position of the wire in the true lumen. The stent graft was successfully deployed from the abdominal aorta to both common iliac arteries without complications. The measured dose area product was 11.46 Gy cm^2^, and the cumulative air kerma was 29.6 mGy, both of which were lower than those typically reported for standard EVAR procedures^6^ and within the acceptable range for pregnant patients.

## Clinical Question 5: Which Delivery Method Is Preferred in This Case?

**Figure 3:**
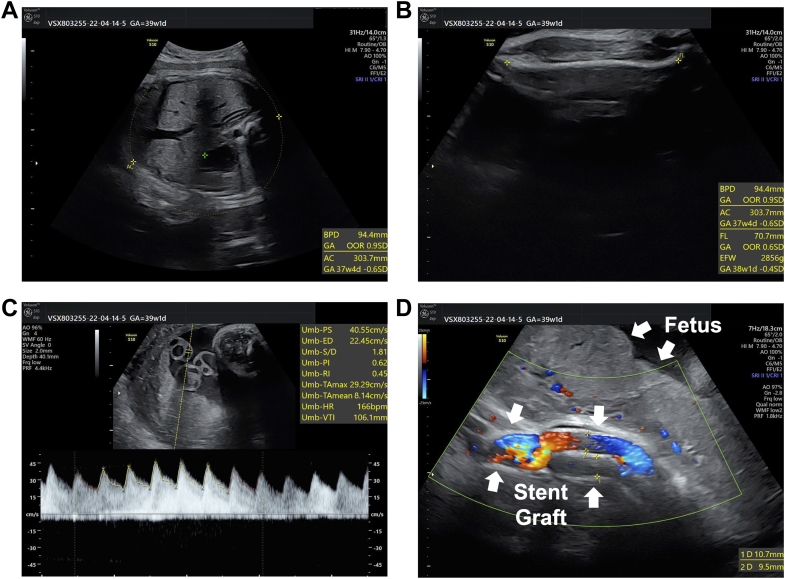
Fetal Ultrasound Findings at 39 Weeks' Gestation (A) Abdominal circumference (303.7 mm). (B) Femur length (70.7 mm). (C) Umbilical artery Doppler tracing obtained on carvedilol 5 mg daily for the patient showed peak systolic velocity 40.6 cm/s, end-diastolic velocity 22.5 cm/s, S/D ratio 1.81, pulsatility index 0.62, resistance index 0.45, and fetal heart rate 166 beats/min. These findings were all within the normal range. (D) Color Doppler image showing the deployed abdominal stent-graft in close proximity to the fetus, with no evidence of compression-induced deformation or graft compromise.

At 39 weeks' gestation, biometric parameters for fetal assessment, including abdominal circumference and femur length, and umbilical artery Doppler indices obtained on carvedilol 5 mg daily were all within normal limits, confirming adequate fetal growth and placental perfusion (Figures 3A to 3C). Color Doppler imaging showed the deployed abdominal stent graft adjacent to the fetus with no evidence of compression-induced deformation or graft compromise (Figure 3D).

The primary goal of controlled delivery in patients with aortic pathology is to minimize cardiovascular stress. In cases where the ascending aortic diameter is between 40 and 45 mm, vaginal delivery with an expedited second stage and epidural anesthesia may be considered to reduce the risk of blood pressure surges. Cesarean delivery should be considered when the aortic diameter exceeds 45 mm and is recommended in patients with vascular Ehlers-Danlos syndrome or in those with acute or chronic AD.^4^ However, the optimal delivery strategy in women with a history of previous aortic intervention has not been clearly established. A previous report documented successful vaginal delivery 6 years after stent-graft placement for AD, suggesting feasibility if the graft remains stable without evidence of dissection or aneurysmal dilatation.^8^ In this case, serial abdominal ultrasound scans after EVAR showed no recurrence of aneurysmal changes or other vascular complications. After multidisciplinary consultation among cardiology, vascular surgery, and obstetrics departments, vaginal delivery was deemed appropriate. She had an uncomplicated vaginal delivery under epidural anesthesia at 39 weeks' gestation with no perinatal adverse events. Five minutes after birth, the neonate's Apgar score was 7; birth length and weight were 49 cm and 3,073 g, respectively, both within normal limits.

## Clinical Question 6: What Points Need to Be Addressed in the Long-Term Follow-Up After Surgery?

**Figure 4:**
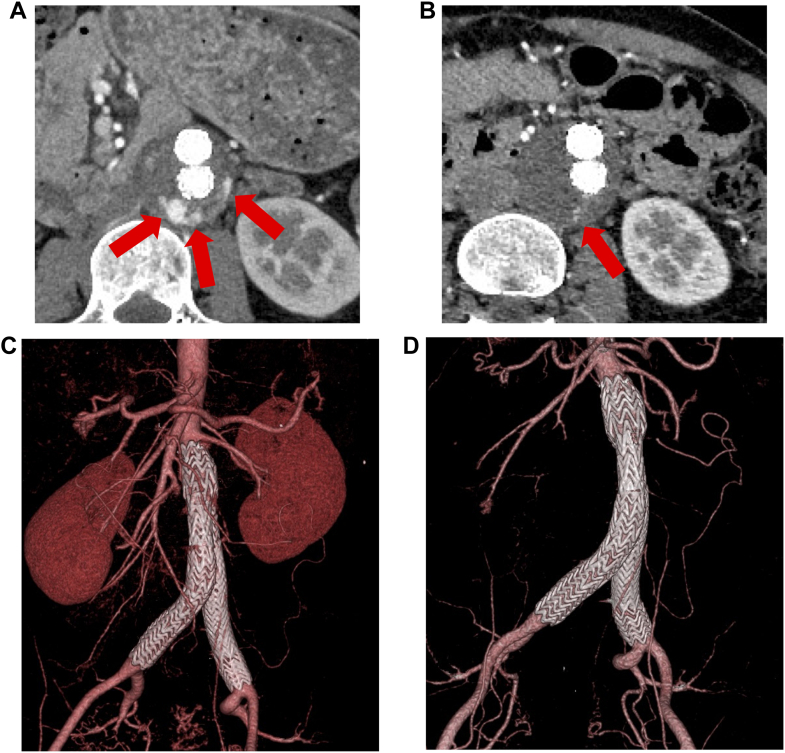
Follow-Up CT After EVAR (A and C) Follow-up computed tomography (CT) at 6 months after endovascular aneurysm repair (EVAR) showed a type II endoleak in the left lumbar artery (red arrow). There was no re-enlargement of the aorta. (B and D) Follow-up CT at 18 months after EVAR showed that the endoleak had decreased in size (red arrow). There was no re-enlargement of the aorta.

In a nationwide registry of 38,003 EVAR procedures performed in Japan between 2006 and 2015, the operative and in-hospital mortality rates were 0.08% and 1.07%, respectively. Renal dysfunction was the most common postoperative complication (2.6%). Endoleaks were reported in 6.7% for type I, 16.6% for type II, and 0.6% for both types III and IV.^9^ In this case, a type II endoleak originating from the left lumbar artery was identified at 6 months post-EVAR (Figure 4A). By 18 months post-EVAR, the endoleak had decreased in size (Figure 4B), and no evidence of recurrent abdominal aortic dilatation was observed (Figures 4C and 4D, Videos 2A to 2C). She continues to be monitored with regular imaging studies and tight blood pressure and heart rate control with carvedilol.

## Clinical Question 7: How Should the Patient's Child Be Managed?

In individuals younger than 18 years of age, genetic testing is indicated when there is a clear clinical indication or a potentially treatable condition is suspected. In such cases, testing is performed with the informed consent of a legally authorized representative and the informed assent of the patient. Given the characteristics of genetic information, such as invariance, predictability, shareability, and ambiguity, the appropriateness of genetic testing in children of aortic aneurysm patients with aortic aneurysm who have not yet developed the disease should be carefully considered.^10^ Although no pathogenic variants have been identified, a hereditary CTD could not be definitively excluded in this case. Therefore, her at-risk child will require long-term clinical surveillance for the possible development of syndromic features or aortic pathology. If such findings are present, improved genetic testing for previously unknown genes and potential risks for genetic disorders should be considered. The patient and her child will undergo ongoing follow-up within a multidisciplinary surveillance framework.

## Conclusions

In the present case, EVAR was an effective treatment option for chronic infrarenal AAA with dissection during pregnancy. In addition, multidisciplinary collaboration among cardiology, vascular surgery, obstetrics, genetics, and pediatrics departments has resulted in favorable outcomes for both the patient and her fetus. The multidisciplinary framework, including early genetic evaluation for CTD assessment, hemodynamic management, regular imaging monitoring, and adjustment of the delivery plan, was essential for the management of aortic disease during pregnancy.

## Funding Support and Author Disclosures

The authors have reported that they have no relationships relevant to the contents of this paper to disclose.

## Appendix

Video 1 CT Videos of Infrarenal AAA With Dissection Before EVAR Computed tomography (CT) scan before endovascular aneurysm repair (EVAR) showed infrarenal abdominal aortic aneurysm (AAA) complicated by dissection extending to the iliac artery; there was no evidence of atherosclerosis. (A) Axial view. (B) Coronal view. (C) Sagittal view.

Video 2 Follow-up CT Videos at 6 Months After EVAR Follow-up CT at 6 months after EVAR showed no evidence of recurrent abdominal aortic dilatation. (A) Axial view. (B) Coronal view. (C) Sagittal view. Abbreviations as in Video 1.
